# A multicenter phase II study of salvage photodynamic therapy using talaporfin sodium (ME2906) and a diode laser (PNL6405EPG) for local failure after chemoradiotherapy or radiotherapy for esophageal cancer

**DOI:** 10.18632/oncotarget.14029

**Published:** 2016-12-20

**Authors:** Tomonori Yano, Hiroi Kasai, Takahiro Horimatsu, Kenichi Yoshimura, Satoshi Teramukai, Satoshi Morita, Harue Tada, Yoshinobu Yamamoto, Hiromi Kataoka, Naomi Kakushima, Ryu Ishihara, Hajime Isomoto, Manabu Muto

**Affiliations:** ^1^ Department of Gastroenterology, Endoscopy division, National Cancer Center Hospital East, Kashiwa, Japan; ^2^ Institute for Advancement of Clinical and Translational Science, Kyoto University Hospital, Kyoto, Japan; ^3^ Department of Therapeutic Oncology, Graduate School of Medicine, Kyoto University, Kyoto, Japan; ^4^ Innovative Clinical Research Center (iCREK), Kanazawa University Hospital, Japan; ^5^ Department of Biostatistics, Kyoto Prefectural University of Medicine Graduate School of Medical Science, Japan; ^6^ Department of Gastrointestinal Oncology, Hyogo Cancer Center, Hyogo, Japan; ^7^ Department of Gastroenterology and Metabolism, Nagoya City University Graduate School of Medical Sciences, Nagoya, Japan; ^8^ Endoscopy Division, Shizuoka Cancer Center, Shizuoka, Japan; ^9^ Department of Gastrointestinal Oncology, Osaka Medical Center for Cancer and Cardiovascular Disieses, Osaka, Japan; ^10^ Department of Gastroenterology and Hepatology, Nagasaki University Hospital, Nagasaki, Japan

**Keywords:** esophageal cancer, photodynamic therapy, salvage treatment, chemoradiotherapy, talaporfin sodium

## Abstract

Photodynamic therapy (PDT) showed promising efficacy for local failure after chemoradiotherapy (CRT) for esophageal cancer. However, PDT required long sun shade period. This study aimed to evaluate the safety and efficacy of PDT using second generation photosensitizer, talaporfin sodium for local failure after CRT. This was the multi-institutional non-randomized phase II study. Patients with histologically proven local failure limited within the muscularis propria after 50Gy or more radiotherapy (RT) for esophageal cancer were eligible. We set the primary endpoint as local complete response (L-CR) per patients. And, secondary endpoints were confirmed L-CR, local progression free survival (L-PFS), progression free survival (PFS), overall survival (OS), L-CR per lesions (Lesion L-CR), and confirmed Lesion L-CR. The PDT procedure commenced with intravenous administration of a 40 mg/m^2^ dose of talaporfin sodium followed by diode laser irradiation at a 664 nm wavelength. 26 eligible patients were enrolled and all were treated with PDT. Twenty three patients with 25 lesions were assessed L-CR after PDT; the L-CR rate per patients was 88.5% (95% CI: 69.8%-97.6%). No skin phototoxicity was observed, and no grade 3 or worse non-hematological toxicities related to PDT were observed. PDT using talaporfin sodium and a diode laser is a safe and curative salvage treatment for local failure after CRT or RT for patients with esophageal cancer.

## INTRODUCTION

Esophageal cancer is the eighth most common cancer worldwide and sixth leading cause of cancer-related death [1]. While neoadjuvant chemoradiotherapy (CRT) or chemotherapy followed by surgery is the standard treatment for resectable esophageal cancer, definitive CRT is still one of the curative treatment options for the patients with un-resectable tumors and those with unsuitable or refusal for surgery. However, locoregional failure after definitive CRT has been the major problem, and its rate was as great as 50-55% [2, 3]. For those patients, salvage surgery is indicated, however, it is associated with a high complication rate (50-77%) and high mortality rate (approximately 15%) [4–7].

Among the patients with locoregional failure after definitive CRT, some patients don't have distant or lymph node metastasis. Such patients have the potential to be cured by local treatment such as endoscopic resection (ER) [8, 9], or photodynamic therapy (PDT) [10–12]. However, ER has limitations for its indication restricted to only mucosal residue due to technical difficulties. PDT is relatively easy to control the technical quality and its effect is expected to deeper residue to submucosal layer. However, PDT using the first generation photosensitizer, porfimer sodium, and excimer dye laser has several problems such as a high occurrence of skin phototoxicity, a long sun shade period requirement as approximately 6 weeks, and the necessity of an expensive and large laser system for excitation.

The second generation photosensitizer, talaporfin sodium (ME2906, Meiji Seika Pharma Co., Ltd., Tokyo, Japan) is rapidly cleared from the skin and require shorter sun shade period (within 2 weeks) [13, 14]. Furthermore, the depth of effect is expected to deeper layer to muscularis propria because the excited wavelength of diode laser (PNL6405EPG, Panasonic Healthcare Co., Ltd., Ehime, Japan) is longer than excimer dye laser (630 nm). Therefore, the second generation PDT is expected as salvage treatment for local failure after CRT for esophageal cancer. After completion of preclinical and phase I study [15–17], we conducted a phase II study to evaluate the efficacy and safety of PDT using talaporfin sodium and a diode laser for local failure after CRT or radiotherapy alone (RT).

## RESULTS

Between November 2012 and December 2013, 26 eligible patients were enrolled and all completed PDT and were included full analysis set. Baseline characteristics of the patients and lesions are shown in Table 1. Twenty one patients were treated with CRT, and the remaining 5 patients were treated with RT. Solitary failure lesions were found in24 of 26 patients, while the other 2 patients had 2 lesions each. All lesions were confirmed as squamous cell carcinoma, and T1b was in 21, and T2 was in 7 lesions, respectively.

**Table 1:** Baseline characteristics of 26 patients and 28 lesions

Characteristics	Number of patients
**Gender**	
**Male**	**26**
**Female**	**0**
**Median age**	**71.5**
**(range)**	**51-86**
**Location**	
**Upper**	**5**
**Middle**	**16**
**Lower**	**7**
**Baseline stage before radiotherapy**	
**T stage**	
**T1b**	**14**
**T2**	**6**
**T3**	**6**
**N stage**	
**N0**	**18**
**N1**	**8**
**Regimen of chemoradiotherapy**	
**Chemoradiotherapy**	**21**
**cisplatin and 5FU**	**11**
**nedaplatin and 5FU**	**3**
**others**	**7**
**Radiotherapy alone**	**5**
**Total dose of radiotherapy (Gy)**	
** ≥50, <60**	**7**
** 60**	**17**
** >60, ≤70**	**2**
**Number of failure lesion**	
** 1**	**24**
** 2**	**2**
**T stage at failure**	
** T1**	**21**
** T2**	**7**
**Failure pattern of lesions**	
** recurrence after achieving CR**	**22**
** residual lesion just after CRT**	**6**

### Treatment flow and efficacy

**Figure 1:**
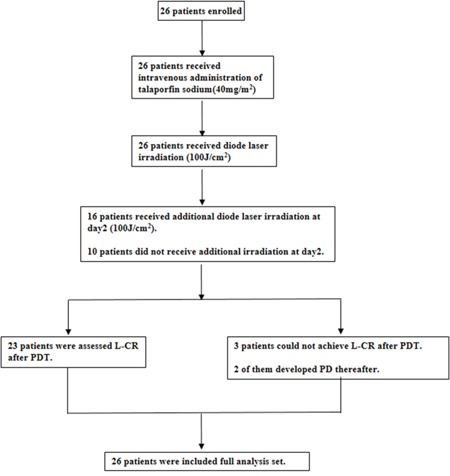
The flow of study 26 eligible patients were enrolled and all completed the intravenous administration of talaporfin sodium followed by diode laser irradiation and were included full analysis set. While 16 patients received additional laser irradiation, the remaining 10 patients did not. 23 patients were assessed local complete response (L-CR), and the other 3 patients could not achieve L-CR after PDT.

**Figure 2:**
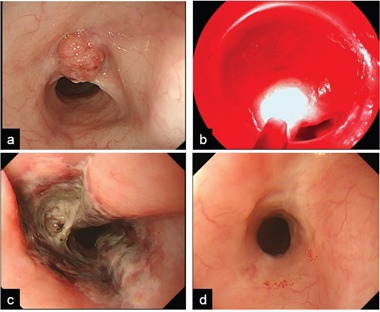
A representative case that was able to achieve CR with PDT **a.** a protruded local failure was present at the anterior wall of middle esophagus, and the estimated depth of the lesion was deep submucosal layer. **b.** After administration of talaporfin sodium, diode laser was exposed to the lesion. **c.** a week after PDT, ischemic change of mucosa was present at laser irradiated site. **d.** two months after PDT, post-PDT ulcer was disappear and scar formation was confirmed and biopsy was negative of cancer cells, therefore complete response was achieved with PDT.

The study flow is summarized in Figure 1. Sixteen patients received additional laser irradiation. Median laser exposure dose at day 1 was 300J (range: 200-800), and median additional laser exposure dose at day2 was 100J (range: 100-300), therefore the median total laser exposure dose was 400J (range: 200-900). The efficacy of PDT is summarized in Table 2. Twenty three patients with 25 lesions were assessed local complete response (L-CR) after PDT (L-CR rate: 88.5%, 95% CI: 69.8%-97.6%), and all patients achieve confirmed L-CR (cL-CR)(Figure 2). The Bayesian posterior probability that the L-CR rate is greater than threshold (15%) was almost 100%. The best response of the other 3 patients was nonCRnon progressive disease (PD), and 2 of them developed L-PD thereafter. All L-CR cases achieved L-CR within 20 weeks after PDT. The L-CR rate and cL-CR rate per lesion was 89.3% (95% CI: 71.8%-97.7%). Moreover, L-CR rate of T1 failure lesions was 100% (19/19, 95% CI: 82.4%-100%), whereas the L-CR rate of T2 failure lesions was 57.1% (4/7, 95% CI:18.4%-90.1%).

**Table 2:** Best response after PDT

n	L-CR	nonCRnonPD	L-PD	NE	CR rate	95%CI
**26 patients**	**23**	**3**	**0**	**0**	**88.5%**	**69.8%-97.6%**
**28 lesions**	**25**	**3**	**0**	**0**	**89.3%**	**71.8%-97.7%**
**T1b lesions (21)**	**21**	**0**	**0**	**0**	**100%**	**83.9%-100%**
**T2 lesions (7)**	**4**	**3**	**0**	**0**	**57.1%**	**18.4%-90.1%**

PDT: photodynamic therapy, L-CR: local complete response, L-PD: local progressive disease

NE: not evaluable, CI: confidence interval All patients and lesions with L-CR could also achieve confirmed CR.

### Safety

The hematological and non-hematological toxicities related to PDT in this study are summarized in Table 3. No skin phototoxicity was observed, and 18 (69.2%) patients were evaluated as non-reactionary with a skin photosensitivity test a week after administration of talaporfin sodium, and all remaining patients were evaluated as non-reactionary within 2 weeks after PDT. Common toxicities related to PDT were esophageal pain in 14 (53.8%), fever in 8 (30.8%). There was no case of treatment related death.

**Table 3:** Toxicities related to PDT

Event	Grade				total (%)
	1	2	3	4	
**Esophageal pain**	**13**	**1**	**0**	**0**	**14 (53.8%)**
**Dysphagia**	**1**	**1**	**0**	**0**	**2 (7.7%)**
**Esophageal stricture**	**2**	**0**	**0**	**0**	**2 (7.7%)**
**Laryngeal pain**	**1**	**0**	**0**	**0**	**1 (3.8%)**
**fever**	**7**	**1**	**0**	**0**	**8 (30.8%)**
**CRP increase**	**20**	**1**	**0**	**0**	**21 (80.8%)**
**Hypoalbuminemia**	**7**	**2**	**0**	**0**	**9 (34.6%)**
**AST increase**	**5**	**0**	**0**	**0**	**5 (19.2%)**
**ALT increase**	**5**	**0**	**0**	**0**	**5 (19.2%)**
**γ-GTP increase**	**3**	**0**	**0**	**0**	**3 (11.5%)**
**Lymphopenia**	**1**	**4**	**2**	**0**	**7 (26.9%)**
**Neutrophil count increase**	**3**	**0**	**0**	**0**	**3 (11.5%)**
**Total bilirubin increase**	**2**	**0**	**0**	**0**	**2 (7.7%)**
**Skin phototoxicity**	**0**	**0**	**0**	**0**	**0 (0%)**

PDT: photodynamic therapy, CRP: C reactive protein, AST: aspartate aminotransferase

ALT: alanine aminotransferase, GTP: glutamyltranspeptidase

### Survival

**Figure 3:**
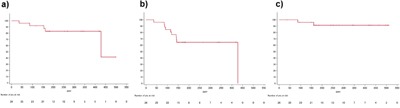
The survival curves in this study **a.** Local progression free survival (L-PFS) curve; 5 events were observed during follow up. **b.** PFS curve; 10 events were observed during follow up. **c.** Overall survival curve; 2 events were observed during follow up.

The survival curves are shown in Figure 3. The median follow up period was 8.4 months (range: 1.2-17), and no patients died from esophageal cancer progression. Two non-CRnonPD patients with PDT developed progression thereafter, and 1 CR patient developed local recurrence approximately 14 months after PDT. The other 2 patients died due to pneumonia at 81 days, and 156 days after PDT. Therefore, 5 events were confirmed for the L-PFS curve at present, and the median L-PFS was 428 days (Figure 3-3a). Total 10 events related to PFS were observed in this study. Three patients developed lymph node or distant metastasis at 91, 115, and 119 days after enrollment, a patient developed intramural metastasis of esophagus at 85 days after PDT and treated with PDT using porfimer sodium. Another superficial esophageal cancer was detected and cured with ER in the other 3 patients. And, 2 patients developed local progression at 87 days and 428 days after PDT, and another patient died with pneumonia at 81 days after PDT same as above. No patients received salvage surgery for recurrence after PDT. Therefore the median PFS was 428 days (Figure 3-3b). As for OS, only 2 events described earlier were observed, and thus the median survival is undefined for this follow up period (Figure 3c).

## DISCUSSION

We herein demonstrated that salvage PDT with talaporfin sodium showed an excellent high local CR rate (88.5%, 95%CI: 69.8%-97.6%) with acceptable safety after failure for definitive CRT. This result is clinically very important because the patients with only local failure at the primary site without metastasis after CRT have a potential to get cured without high-risk salvage surgery.

In this study, we adopted the CR rate rather than the response rate as a primary endpoint. As PDT is a local treatment, it can provide treatment effect only for the local site. In our previous study using porfimer sodium, patients who could achieve CR at the primary site survived longer [18]. Therefore, we believe that local control, especially the CR rate at the primary site, has clinical significance in salvage PDT strategy. Regarding the outcomes of CRT, the survival benefits of achieving CR were also clarified [19, 20]. In contrast, the survival of the patients who could not achieve CR was quite dismal, and most died within 1 year [20]. Furthermore, patients who could not achieve CR with CRT developed progressive disease at a median timeframe of approximately 1 month [21], and that might lead to dysphagia due to obstruction with progressive primary disease. Therefore, achieving CR at primary site is clinically important not only for survival but also for patients’ quality of life.

As for safety, the toxicity of PDT using talaporfin sodium and the laser was quite mild. The most severe toxicities related to PDT are esophageal perforation at the laser irradiated site. In our previous study of PDT using porfimer sodium, we experienced one treatment death due to gastrointestinal hemorrhage that might be caused by an esophageal-aortic fistula at the laser irradiated site [9]. Therefore, we excluded lesions that potentially involved the aorta before CRT, and there were no cases of perforation or treatment related death in present study. While in our prior study of PDT using porfimer sodium, we experienced 32% skin phototoxicity with at least a month of sun shade period after administration of porfimer sodium [9]. In the present study, there were no cases of skin phototoxicity, even with 2 weeks sun shade period after administration of talaporfin sodium, and this might be one of the greatest strengths of this new generation photosensitizer.

There are several limitations in this study. The study was a single arm trial and did not compare the efficacy to the standard treatment. However, at this moment, there is no established standard treatment for local failure after CRT for patients with esophageal cancer. Furthermore, it might be quite difficult to carry out a randomized trial of PDT versus salvage surgery because of the difference of invasiveness. A randomized trial of PDT versus systemic chemotherapy is also difficult because achievement of CR with systemic chemotherapy is quite difficult [22–26]. Taken all together, the high L-CR rate (88.5%) at the primary site of this new generation PDT for local failure after CRT should be considered of value as a treatment option. The follow up period of this study is insufficient. One-year OS of the patients in this study was 91.4% (95%CI: 69.7%-97.8%). Therefore, short-term survival benefit of salvage PDT could be expected. Further follow up study with same subjects is now ongoing to confirm the long term survival benefit.

In conclusion, PDT using talaporfin sodium and a diode laser is a safe and potentially curative salvage treatment for local failure after CRT or RT for patients with esophageal cancer.

## MATERIALS AND METHODS

### Study design

This study was an investigator initiated multi-institutional non-randomized open label phase II study. The study complied with the Good Clinical Practice Guideline for drugs and medical devices, and the 2008 Declaration for Helsinki. The study protocol was approved by the institutional review board of all 7 participating hospitals. The study was registered with the University Hospital Medical Information Network Clinical Trials Registry (UMIN000009184).

### Eligibility

The eligibility criteria were as follows: 1) local failure after CRT or RT (≥50Gy); 2) one week or longer from the last treatment for esophageal cancer, and any previous treatment is acceptable, except for PDT with talaporfin sodium or porfimer sodium; 3) histologically proven local failure, and either not candidates for salvage surgery or having a physical status that would make the surgery intolerable; 4) salvage ER not indicated due to incurability; 5) no invasion to the cervical esophagus; 6) lesions limited to within the muscularis propria; 7) longitudinal lesion length of 3 cm or shorter and 1/2 the circumference of the lumen or less; 8) no more than 2 lesions; 9) age ≥ 20 years old; 10) Eastern Cooperative Oncology Group performance status (ECOG-PS): 0-2; 11) adequate organ functions (white blood cell counts ≥2000 /mm^3^, hemoglobin >8.0g/dL, platelet count ≥75000/mm^3^, serum total bilirubin level ≤3.0 mg/dL, both alanine transferase and aspartate aminotransferase <100IU/L), 12) provision of written informed consent. The exclusion criteria were as follows: 1) distant organ or lymph node metastasis that required systemic chemotherapy; 2) other active cancers, except for early cancers that are expected to be cured with local treatment such as ER, or cancers that do not require systemic treatment such as chemotherapy; 3) significant cardiovascular diseases (uncontrolled hypertension, myocardial infarction, unstable angina, congestive heart failure), uncontrolled diabetes mellitus, severe liver cirrhosis, severe renal disorder; 4) systemic infection requiring antibiotics; 5) inability to comply the sun shade restrictions; 6) additional PDT just after salvage ER for local failure; 7) baseline lesions before CRT that were judged to involve the aorta; 8) porphyria, 9) preexisting condition of sun photosensitivity; 10) previous treatment with PDT using porfimer sodium or talaporfin sodium; 11) for women, pregnancy or lactation, or unwillingness to use contraception; 12) severe bleeding or shock status; 13) bleeding tendency; 14) current participation or prior participation within 3 months in other clinical trials for unapproved and off-label drugs or medical devices; and 15) patients who judged by the investigator that enrollment was inappropriate.

### Staging

Clinical stage was determined according to the TNM classification of the International Union Against Cancer 7^th^ edition [27] and the *Japanese Classification of Esophageal Cancer*, 10^th^ edition, revised version [28]. Clinical T stage was evaluated by endoscopy, endoscopic ultrasound (EUS), and computed tomography (CT) of the chest. Clinical N and M stages were evaluated by EUS and CT of the neck, chest and abdomen.

### Procedure

**Figure 4:**
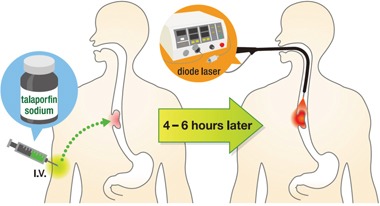
Illustration of PDT procedure

The PDT procedure commenced with intravenous administration of 40 mg/m^2^ dose of talaporfin sodium followed by laser irradiation at a 664 nm wavelength after 4 to 6 hours after administration (Figure 4). The diode laser was delivered via frontal light distributor through the operative channel of endoscope. The plastic attachment was fitted in front of the scope to keep the distance between the tip of the scope and lesion. The fluence of diode laser was set at 100J/cm^2^ with a fluence rate of 150 mW/cm^2^. If the lesions were larger than 1 cm^2^, multiple treatment fields were overlapped to cover the whole lesion. Endoscopic observation was mandatory at the next day, and if an obvious residual tumor was founded, additional laser irradiation was recommended. “Obvious residual tumor” refers to 1) the presence of a residual submucosal tumor-like protruded component, 2) the presence of neoplastic mucosa or ulcer, and 3) the absence of edematous mucosa with redness or dark blue discoloration due to incomplete irradiation.

### Follow up and evaluation

All patients were instructed to avoid direct sun exposure, and to stay in a room maintained at 500 lux or less for 2 weeks after PDT. A skin photosensitivity test was performed every week after administration of talaporfin sodium, and discharge was allowed at day 15 if the skin photosensitivity and other adverse events related with PDT disappeared. Patients were assessed with physical examinations, measurements of hematological and biochemical variables in the blood, chest radiograph studies, and endoscopic observation at least once a week until 28 days after PDT. Adverse events and toxicity were evaluated and graded according to Common Terminology Criteria for Adverse Events (CTCAE) version 4.0. [29]

### Efficacy

Clinical effect at the laser irradiated site was made by endoscopic examination with biopsy at day 22 and 29 after PDT, and every 2 weeks during the second month, and every month thereafter for 6 months after PDT. The local efficacy was classified with endoscopic evaluation as L-CR, local progressive disease (L-PD), local nonCRnonPD (L-nonCR/nonPD), and not evaluable (NE) at each evaluation. The criteria of L-CR was as follows; 1) no residual tumor was observed, 2) disappearance of post-PDT ulcer and scar formation was confirmed, and 3) histologically disappearance of cancer cells. cL-CR was defined as 4 weeks or longer continuation of L-CR status. Endoscopic re-evaluation was performed by a central evaluation committee consisting of 3 experienced endoscopists who were independent to this study. The final efficacy outcome was determined by this committee. Conversely, the criteria of L-PD were as follows: 1) definitive progression compared with the lesion before enrollment, or 2) recurrence after achieving L-CR with PDT. And, when neither L-CR nor L-PD criteria were not met, lesions were classified as L-nonCR/nonPD. Furthermore, when endoscopic examination and tissue diagnosis by biopsy cannot be performed for some reason or when the lesion cannot be classified as L-CR, L-PD, or L-nonCR/nonPD, lesions were classified as NE. CT of the neck, chest and abdomen was performed every 3 months after PDT. Lymph node metastasis was clinically diagnosed if the lymph node was visible and larger than 10 mm.

### Endpoints

The proportion of subjects whose best effect in the overall local therapeutic efficacy is L-CR is referred to as the L-CR rate per patients. And L-CR rate per patients was the primary endpoint of this study. And the proportion of lesions in which the best effect in the lesion local therapeutic efficacy is L-CR is referred to as the L-CR rate per lesion, and L-CR rate per lesion and confirmed L-CR rate per lesion were the secondary endpoints. And other secondary endpoints were local progression free survival (L-PFS), progression free survival (PFS), overall survival (OS).

### Statistics

The threshold L-CR has been set to 15% for T1 and T2 patients because the clinical significance would be limited unless the L-CR rate is confirmed to be more than this threshold. The expected L-CR rates for T1 and T2 patients have been set to 75% and 40%, respectively. A required sample size of 25 patients is calculated using Bayesian approach based on prior predictive distributions in single arm study [30], in which the uncertainty in T1:T2 ratio in enrolled patients is also taken in to account by prior beta distribution with mode of 0.69 (9/13). However, ranges less than 0.24 and greater than 0.76 are truncated here, given that at least some T1 and T2 patients will be enrolled in this clinical trial. Treatment efficacious is declared if the Bayesian posterior probability that the L-CR is greater than the threshold value of 15% exceeds λ = 97.5%. Under the abovementioned settings, this sample size retains a one-sided alpha of 2.5% and power of 90% also in a frequentist analysis and interpretation, that is, this trial is designed to assure valid analyses in either traditional frequentist or Bayesian approaches. L-PFS was measured from the date of enrollment to the first date of confirmation of L-PD or death from any cause. If any events were not observed, the period was censored at the date of last endoscopic evaluation. And, PFS was measured from the date of enrollment to the first date of confirmation of L-PD or new metastasis or progression in a lymph node or a distal organ, or death from any cause. If any events were not observed, the period was censored at the date of last imaging test evaluation or physical examination. And, OS was measured from the date of enrollment to death from any cause. If any event was not observed, the period was censored at the date of last day on which the patient's survival has been confirmed. All authors had access to the study data and had reviewed and approved the final manuscript.
